# Epidemiological Evolution of Lung Cancer in the South of Spain from 1990 to 2010

**DOI:** 10.3779/j.issn.1009-3419.2018.01.05

**Published:** 2018-01-20

**Authors:** Alberto Caballero Vázquez, Ana Dolores Romero Ortiz, Jose Manuel González de Vega San Román, Raimundo García del Moral, Bernardino Alcázar Navarrete

**Affiliations:** 1 Interventional Pulmonology Unit, Universitary Hospital Virgen de las Nieves, Granada, Spain; 2 Department of Pathology, Complejo Hospitalario Universitario de Granada, Spain; 3 High Resolution Hospital of Loja, Granada, Spain

**Keywords:** Lung neoplasms, Tobacco, Histology

## Abstract

**Background:**

Changes in lung cancer has been characterized by the increase of cases among women and the increase in adenocarcinomas among other histological subtypes.

**Methods:**

Descriptive analysis of cases diagnosed with lung cancer in Hospital Virgen de las Nieves (Spain) from 1990 to 2010, based on five variables (age, sex, smoking, histology and pathological anatomy). The study establishes associations between these variables and compares the results with the literature.

**Results:**

2, 026 patients were diagnosed with lung cancer in this period; 1, 838 were males (90.7%) and 188 women (9.3%); 1, 892 patients (93.4%) were smokers or ex-smokers and 134 (6.6%) had never smoked; the most frequent non-small cell histology types were squamous cell carcinoma and adenocarcinoma and it was the most frequent neoplasia in women and were associated with a lower tobacco consumption.

**Conclusion:**

The large majority of lung cancer cases is associated with a history of smoking tobacco and there are histopathological differences according to gender and cumulative tobacco smoke load.

Since the beginning of the lung cancer epidemic, the incidence in women has been less than in men, although, at world level in the last few years there has been a trend towards them both becoming the same, as such that in the USA, the ratio is nearly 1:1^[1]^. In Spain the male/female ratio, although much lower than years ago, is still high^[2]^.

Among the risk factors described for lung cancer, smoking tobacco is the main cause, being responsible for more than 90% of cases, not only directly, but indirectly (passive smoking) and in association with other substances (asbestos, radon, silica, *etc*.)^[3]^. Cigarette smoking is the main cause of the lung cancer histological types, particularly the squamous cell carcinoma and small-cell carcinoma subtypes^[4, 5]^. Studies carried out in the last few years have shown that the most common histological type in the USA is currently adenocarcinoma^[6]^. In general, this lineage is the one that is less associated with smoking, as such that the proportion of adenocarcinomas among non-smokers varies between 40% and 76%. On the other hand, a heavy accumulation of tobacco smoking is a preferential factor for developing squamous cell and small-cell type carcinoma^[7]^.

The main aim of this study is to present a descriptive analysis of the sociodemographic and clinical profile of patients diagnosed with lung cancer in the Technical Diagnostic Unit of Pulmonology Department of the Hospital Virgen de las Nieves (Granada, Spain) over a period of 20 years, from January 1990 until 31 December 2010.

Another aim of the study is to analyse the relationship between the different sociodemographic and clinical variables included.

Due to the considerable importance of this disease as regards the associated mortality and morbidity, we consider it appropriate to conduct a study from a large register of cases to determinate the current situation of lung cancer in our health area.

## Methods

### Sample

It is a retrospective descriptive study from a case register made up of all the patients diagnosed histologically with lung cancer in the Technical Diagnostic Unit of the Chest Diseases Department of the Hospital Virgen de las Nieves, in Granada (Spain) between 1 January 1990 and 31 December 2010, as set out in the register of the Unit itself and in the Tumours Register of the Hospital. The Hospital Virgen de las Nieves covers the northern area of the province of Granada, with a total of 439, 035 inhabitants. A total of 2, 026 patients were diagnosed with lung cancer between 1 January 1990 and December 2010, and these individuals make up our final sample included in the present study.

### Measurements

Five variables were taken into account, two of them quantitative (age and smoking habits), with the remaining three being qualitative (gender, histology, and histopathology). As regards smoking habits, the cumulative tobacco load index "packs/year index", defined as the result of multiplying the number of packs that a subject smokes per day by the years that this amount has been smoked^[8]^. As regards the histology, two large groups were differentiated: small cell cancer and non-small cell cancer. Four categories were established using the pathological anatomy result: adenocarcinoma, squamous cell carcinoma, other non-small cell carcinomas (we include here carcinomas that, being non-small cell, had not been able to be classified within the previous sub-groups for several reasons, such as lack of material that prevented a more exact evaluation of the disease, absence of definitive morphological elements, impossibility to obtain more bronchial material due to technical or clinical problems, *etc*.), and small cell carcinoma itself.

### Statistical analysis

In a first phase, a general descriptive analysis was performed on the variables included in the study, which included a description of the means (and their corresponding standard deviations) in the case of continuous variables such as age and smoking habit, a description of the absolute and relative (percentage) frequencies, in the case of qualitative variables, such as gender, histology type, and the histopathology of the carcinoma. In a second phase, and with the objective of determining the possible existence of an association between categorical variables, contingency tables were constructed, and the Chi square test used, setting a statistical significance level of *P* < 0.05. The comparison of the means between groups is made using the ANOVA test and the Levene test of homogeneity of the variances. When necessary, due there not being a normal distribution of the variables analysed (according to the *Kolmogorov-Smirnov* test), non-parametric tests (such as the *Kruskall-Wallis* and *Mann-Whitney* tests) were used to analyse the possible relationship between variables. The SPSS 15.0 statistics program was used.

## Results

### Descriptive analysis

A total of 2, 026 patients were diagnosed with lung cancer between 1 January 1990 and 31 December 2010 in the Technical Diagnostics Unit of the Hospital Virgen de las Nieves, Granada. Of these, 1, 838 were males (90.7%) and 188 (9.3%) were women (approximate ratio, 10:1), with a mean age of (65.38±9.7) years in males and (62.15±12) years in females. As regards smoking, 1, 892 patients (93.4%) were smokers or ex-smokers at the time of diagnosis, and 134 (6.6%) had never smoked. A histological diagnosis of non-small cell cancer was obtained in 1, 628 (80.4%) cases, and small cell carcinoma in 398 (19.6%) patients. The most frequent non-small cell histology types were squamous cell carcinoma and adenocarcinoma, with 1, 061 (52.4%) and 281 (13.9%) cases, respectively. Small cell or microcytic cancer was diagnosed in 401 patients (19.8%). The 283 patients (14%) that were diagnosed with poorly/moderately differentiated non-small cell cancer are included in this study within the sub-group 'other non-small cell carcinomas' (Table 1).

**1:** Descriptive variables of the total sample

Variables	Data	
Gender		
Male	1, 838 (90.7%)	
Female	188 (9.3%)	
Average age (yr)	63.76±10.8	
Smoker or ex smoker		
Yes	1, 892 (93.4%)	
No	134 (6.6%)	
Histology		
Small cell lung cancer	401 (19.8%)	
Non small cell lung cancer	1, 625 (80.2%)	
Pathological anatomy		
Squamous cell carcinoma	1, 061 (52.4%)	281
Adenocarcinoma	(13.9%)	
Other non-small cell carcinomas	283 (14.0%)	
Small cell lung cancer	401 (19.8%)	

### Analysis of the relationship between variables

#### Smoking habit according to gender

When evaluated by gender, it was observed that 98.2% of the men were smokers or ex-smokers, and 1.8% had never smoked. Of the women, 46.3% were smokers or ex-smokers and 53.7% non-smokers (*P* < 0.000, 1). The mean cumulative packs/year index for men was (60.13±27.5), and for females, (10.90±18.45) (*P* < 0.000, 1).

#### Histology according to gender

Our results show that there is a statistically significant relationship between the histology carcinoma type and gender. In particular, small cell type tumours were more common in men than in women.

#### Pathological anatomy according to gender

Squamous cell carcinoma was the most common cancer in males, with a total of 1, 002 cases (54.51%). Adenocarcinoma was diagnosed in 208 patients (11.31%), small cell carcinoma in 385 (20.94%), and poorly or moderately differentiated non-small cell carcinoma in 243 (13.22%). Adenocarcinoma was the most frequent malignant tumour in women. Thus, of the 188 diagnosed with lung cancer, 73 (38.82%) had an adenocarcinoma, 59 (31, 38%) a squamous cell carcinoma, 16 (8.51%) a small cell carcinoma, and 38 (20.21%) a poorly or moderately differentiated non-small cell carcinoma ('other non-small cell carcinomas')(Table 2). Our results showed that there was a statistically significant difference between men and women as regards the histopathology type (Chi-square=131.763, *P* < 0.001).

**2:** Pathological anatomy according to gender

	Men	Women
Squamous cell carcinoma	1, 002 (54.5%)	59 (31.4%)
Adenocarcinoma	208 (11.3%)	73 (38.8%)
Other non-small cell carcinoma	243 (13.2%)	40 (21.3%)
Small cell	385 (20.9%)	16 (8.5%)

#### Histology type according to smoking habit

Statistically significant differences were found between different histology types (small cell and non-small cell) and smoking habits (number of packs/year), with a larger consumption of tobacco being observed in patients with small cell cancer (54 packs-years in non small cell cancer and 58 packs-years in small cell cancer, *P*=0.037).

#### Pathological anatomy - Smoking habit (overall)

Statistically significant differences were also seen between the number of packs/year and the different histopathology subtypes, with the packs/year index being higher in the squamous cell and small cell carcinomas subtypes, and a lower tobacco consumption in the adenocarcinoma subtype (*P* < 0.001; Table 3).

**3:** Pathological anatomy - Smoking habit (overall)

	Mean packs-year index	Standard deviation
Squamous cell (*n*=1, 061)	60.045	20.541
Adenocarcinoma (*n*=281)	39.089	32.697
Other non-small cell (*n*=283)	50.349	31.274
Small cell (*n*=401)	59.160	28.239

#### Pathological anatomy - Smoking habit (stratified analysis by gender)

On stratifying these data according to gender, it was observed that, although the packs/year smoked by women is clearly lower than that in men, the relationship between squamous cell and small cell tumours and a greater smoking habit is the same in both sexes (Table 4), while the adenocarcinomas are associated with a lower tobacco consumption

**4:** Pathological anatomy - Smoking habit (stratified analysis by gender)

P. Anatomy	Mean packs/year		
	Men	Women	
Squamous cell	62.599	16.678	U=5, 344.500; *P* < 0.001
Adenocarc	50.587	6.329	U=1, 299.500; *P* < 0.001
Other non-small cell	56.959	8.079	U=453.000; *P* < 0.001
Small cell	60.904	17.188	U=671.000; *P* < 0.001

## Discussion

Lung cancer is the main cause of death by cancer in the world, and tobacco is the most important risk factor for its development, and is implicated in 90% of cases^[9-11]^. Several epidemiological studies on lung cancer in Spain have been published over the last few years^[12, 13]^, in which it has been shown that the percentage of males affected continues to be higher than that in women. However, it is important to highlight the increase in incidence and mortality rates among the latter. In our study, the percentage of males with lung cancer was 90.7%, compared to 9.3% among women, and the mean age of patients at the time of diagnosis was 65 years in males and 62 years in women, similar data to those observed by other authors from our country and similar to the majority of European case series^[14]^.

As regards smoking according to gender, in our study we observe that 46.3% of women were smokers compared to 98.2% of male smokers. These data contrast with those at national level, where figures around 31.6% in males and 21.5% in women are mentioned, as reflected in the National Statistics Institute in the National Health Survey of 2006. On the other hand, and as has already been reported by other authors, we found that the lung cancer in non-smokers is more frequent in women than in men^[15]^. In our study we found that women had a lower cumulative tobacco consumption than men (10.90 compared to 60.13), as is observed in some other studies^[16]^. As regards the histopathology types, the most frequent subtype was squamous cell carcinoma, with 1, 061 (52.4%) cases, followed by small cell carcinoma with 401 (19.8%). These data contrast with that found in many studies, where we can observe that squamous cell carcinoma continued being the most frequent, followed closely by adenocarcinoma^[17]^, and even this latter is most frequent in North American series. Perhaps we can partly explain this phenomenon for the still relatively small proportion of women in our series, since adenocarcinoma is the most frequent subtype in this gender^[18]^. As regards histopathology subtypes according to smoking habits, it was observed that those most associated with a higher tobacco load were squamous cell carcinoma (60.04 packs/year), very closely followed by small cell carcinoma (59.15 packs/year), with adenocarcinoma being the least associated with tobacco consumption (39.08 packs/year). These findings are similar to those published in the large majority of the literature^[19]^. As mentioned before, the elevated percentage of adenocarcinoma in women with a low consumption of tobacco in our study should oblige us to continue study in-depth the importance of the other carcinogens besides those in tobacco smoke. Similarly, molecular and/or hormonal factors may have an influence on the histology differences between both sexes.

## Conclusions

It may be concluded that lung cancer is still a highly significant condition, with the large majority of cases associated with a history of smoking tobacco, and with histopathological differences according to gender and cumulative tobacco smoke load. Greater knowledge of this disease would enable us, in the near future, to individualise the therapeutic approach to lung cancer by developing increasing more personalised treatments for each patient.

## Conflict of interest

None.

## Acknowledgment

This article is associated with the doctoral thesis of the main author, linked to the University of Granada (UGR).

## References

[1] López-Abente G, Pollan M, Aragoneses N (2004). Situación del cáncer en España: incidencia. Anales Sis San Navarra.

[2] Estrada Trigueros G, Comeche L, López Encuentra A (2007). Carcinoma broncogénico 2000-2001. Características y supervivencia global. Arch Bronconeumol.

[3] Egleston BL, Meireles SI, Flieder DB (2009). Population-based trends in lung cancer incidence in women. Semin Oncol.

[4] Simonato L, Agudo A, Ahrens W (2001). Lung cancer and cigarette smoking in Europe: an update of risk estimates and an assessment of inter-country heterogeneity. Int J Cancer.

[b5] 5Hrubec Z, McLaughlin JK. Former cigarette smoking andmortality among US veterans: a 26-year follow-up, 1954-1980. En: Burns D, Garfinkel L, Samet JM, eds. Changes in cigarette-related disease risks and their implication for prevention and control. Bethesda, MD: US Government Printing Office, 1997: 501-530.

[6] Charloux A, Quoix E, Wolkove N (1997). The increasing incidence of lung adenocarcinoma. Reality or artefact? A review of the epidemiology of lung adenocarcinoma. Int J Epidemiol.

[7] Tyczynski JE, Bray F, Parkin DM (2003). Lung cancer in Europe in 2000; epidemiology, prevention and early detection. Lancet Oncol.

[8] Barberá JA, Peces-Barba G, Agustí AG (2001). Guía clínica para el diagnóstico y el tratamiento de la enfermedad pulmonar obstructiva crónica. Arch Bronconeumol.

[9] Hirsch FR, Matthews MJ, Aisner S (1988). Histopathologic classification of small cell lung cancer: changing concepts and terminology. Cancer.

[b10] 10US Department of Health and Human Services. Women and smoking: a report of the surgeon general. Washington, DC: Public Health Service. Office of the Surgeon General; 2000.http://europepmc.org/abstract/MED/11906680

[11] Hernández JR, Tapias JA, Moreno P (2004). Lung cancer incidence in the province of Avila, Spain in 2002 and decade-long trends. Arch Bronconeumol.

[12] Montero C, Rosales M, Otero I (2003). Lung cancer in the health care area of A Coruña (Spain): incidence, clinical approach and survival. Arch Bronconeumol.

[13] Rezola-Solaun R, Sanzo-Ollakarizketa JM (1999). The incidence, trend and survival in lung cancer by histological type in Gipuzkoa (1983-1992). Rev Clin Esp.

[14] Mäkitaro R, Pääkkö P, Huhti E (1999). An epidemiological study of lung cancer: history and histological types in a general population in northern Finland. Eur Respir J.

[15] Sun S, Schiller JH, Gazdar AK (2007). Lung cancer in never smokers-a different disease. Nat Rev Cancer.

[16] Belani CP, Marts S, Schiller J (2007). Women and lung cancer: epidemiology, tumor biology and emerging trends in clinical research. Lung Cancer.

[17] Santos-Martínez MJ, Curull V, Blanco ML (2005). Lung cancer at a university hospital: epidemiological and histological characteristics of a recent and a historical series. Arch Bronconeumol.

[18] Hoffman PC, Mauer AM, Vokes E (2000). Lung cancer. Lancet.

[19] Alberg AJ, Ford JG, Samet JM (2007). Epidemiology of lung cancer. ACCP Evidence -Based Clinical Practice Guidelines (2^nd^ edition). Chest.

